# Cap snatch prevention: a novel approach to tackle influenza viruses

**DOI:** 10.1038/s41392-023-01474-9

**Published:** 2023-05-08

**Authors:** Konstantin M. J. Sparrer, Frank Kirchhoff

**Affiliations:** Institute of Molecular Virology, Ulm University Medical Center, Ulm, Germany

**Keywords:** Infectious diseases, Drug development

**Fig. 1:**
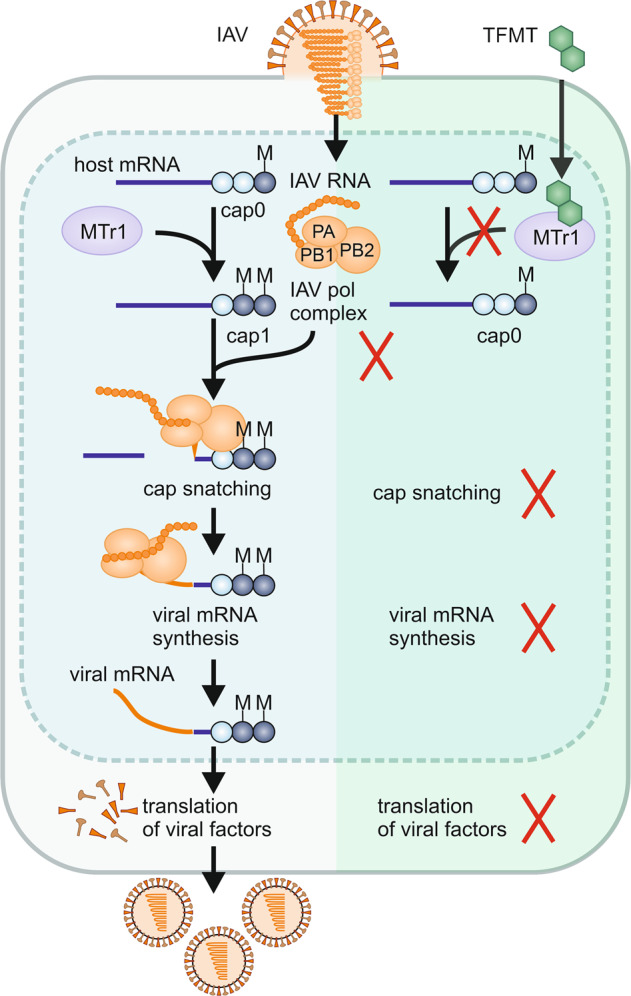
The MTr1 inhibitor TFTM prevents IAV and IBV cap-snatching and thus restricts viral replication. Host 5’cap0-mRNAs are matured to cap1 mRNAs by the cellular Cap-specific mRNA (nucleoside-2’-O-)-methyltransferase 1 (MTr1). The IAV/IBV viral polymerase complex PB2/PA/PB1 selectively recognizes MTr1 modified cap1 structures, cleaves them off the cellular mRNA and uses them as primers for the synthesis of viral mRNAs (“cap-snatching”). The camouflaged viral mRNAs are then readily translated into viral proteins to eventually promote viral progeny. Trifluoromethy-tubericin (TFMT) inhibits MTr1 and thus prevents cap-snatching by IAV and IBV and inhibits viral replication

A recent study published in Science^1^ by Tsukamoto and colleagues shows that a derivative of tubercidin, a natural product from Streptomyces, selectively inhibits influenza A and B viruses (IAV and IBV, respectively). The compound targets the host RNA methyltransferase MTr1, and thus prevents viral “cap snatching” (Fig. 1). These results may help to develop novel drugs that target viral host dependency factors and are thus less likely to induce viral resistance.

Influenza remains a global health threat affecting millions of people and causing hundreds of thousands of deaths each year. Despite ongoing surveillance, it is impossible to predict which zoonotic virus strains may emerge and vaccine efficiency in preventing illness is typically just ~40–60%. Drugs targeting the viral neuraminidase (NA) or ion channel (M2) have been approved for clinical use.^2^ However, influenza viruses evolve rapidly (anti-genic drift)^2^ and many circulating strains of IAV and IBV are resistant against existing therapeutic agents. Therefore, there is a dire need for novel therapeutic strategies against influenza. In addition, coinfection of birds, pigs or humans infected with different IAV strains may result in gene reassortments (anti-genic shift) leading to the emergence of novel viral strains that are resistant to existing prophylactic and therapeutic measures. Although antigenic shift is rare, its impact can be devastating as seen in the 1918 Spanish flu pandemic. Importantly, therapeutics targeting cellular factors critical for viral replication are less prone to viral resistance.^3^

Cellular mRNAs are initially 5’terminally capped by 7-methylguanosine or 2,2,7-trimethyl-guanosine (cap0) and subsequently methylated at the first nucleotide by the cellular host 2′-O-ribose methyltransferase 1 (MTr1) resulting in the mature cap1 structure. This ensures recognition by the ribosome, stabilizes the mRNA, and prevents recognition by innate immune sensors. Successful viruses mimic the mature cap1 structure on their viral mRNAs to avoid immune activation and ensure efficient replication. Some viruses encode their own methyltransferases to modify the 5’cap. In contrast, Bunya- and Orthomyxoviruses including influenza viruses “steal” the mature cap1 structure from host cell mRNAs (Fig. 1).^4^ During “cap snatching” the viral polymerase containing PB1, PB2 and PA cleaves mature cellular mRNAs downstream of the 5’cap1 structure and uses the snatched 5’cap1 RNA as a primer for the nascent viral mRNA. Of note, the PB2 subunit of the IAV polymerase has previously been recognized as a promising drug target.^5^ However, no inhibitors of the cellular MTr1 enzyme have been reported.

To evaluate whether MTr1 can be exploited in host-targeting anti-influenza approaches, Tsukamoto and colleagues first generated cell lines lacking MTr1 expression.^1^ In these cells, replication of IAV and IBV was strongly attenuated and rescued by ectopically expressing functional but not catalytically inactive MTr1. Notably, only viral mRNA but not cellular mRNA expression was impaired. MTr1 KO restricted various IAV and IBV strains but had little if any effect on other cap-snatching viruses, like Influenza D viruses or *Bunyaviridae*. After an in silico screen of 5597 compounds and molecular docking studies using the MTr1 crystal structure (PDB ID: 4N49) they identified tubercidin, a naturally occurring adenosine analog from *Streptomyces*, as a putative binding partner. In vitro experiments confirmed that tubercidin inhibits MTr1 through interaction with its S-adenosyl-L-methionine binding pocket. Since tubercidin is cytotoxic, the authors performed an elegant series of experiments using more than 100 tubercidin-related compounds to identify trifluoromethyl-tubercidin (TFMT) as an effective antiviral agent that targets MTr1 with no apparent in vitro toxicity.

The authors show that TFMT inhibits replication of different IAV and IBV strains, including seasonal strains, in primary human bronchial lung cells in vitro and protects human lung explants ex vivo against infection and virus-induced pathology. Although TFMT was less effective in a mouse cell line compared to human cells (IC_50_ 7.7 µM vs 0.3 µM) it still prevented IAV infection induced weight loss in mice and reduced the levels of viral replication in vivo.

MTr1 inhibition by TFMT treatment may increase the levels of immature cap0 RNAs known to be sensed by RIG-I to induce antiviral IFN responses. Thus, TFMT treatment may inhibit influenza viruses by inducing innate antiviral factors. However, in a series of experiments involving pharmacological inhibition of innate immune signaling and genetic KO of sensors, Tsukamoto and colleagues demonstrated that TFMT inhibits viral replication directly by affecting its cap-snatching activity and not through immune modulation. Using structural modelling, they show that the absence of the additional methyl group in cap1 attached by MTr1 would prevent binding to PB2. Of note, depletion of alternative methyltransferases does not affect IAV or IBV replication. Thus, cap-snatching of IAV and IBV specifically requires MTr1-modified caps but not caps processed by other methyltransferases. TFMT acts synergistically with approved anti-influenza drugs and remained active against a baloxavir marboxil-resistant IAV mutant.

Several cap-snatching viruses do not require MTr1 and are baloxavir- and marboxil-resistant raising the possibility that IAV and IBV may also be able to develop resistance to TFMT. However, mutant IAV constructs containing various changes in the cap-binding site of PB2 generally required MTr1 for replication. Thus, determinants of cap-snatching seem conserved, suggesting resistance against MTr1 inhibitors development may be difficult. However, given the versatility of IAV and IBV and alternative cap-snatching mechanisms in closely related viruses like influenza D virus future development of resistance cannot be excluded.

This study of Tsukamoto and colleagues impressively demonstrates how basic research of viral properties may be translated to anti-viral drug development. While the results of the study are promising, some limitations are noteworthy. The IC_50_ of TFMT is relatively high and MTr1 plays an important role in cellular mRNA maturation and avoiding immune detection. The mice analyzed in this study received the first dose of TFMT within an hour after virus exposure and were only treated over two days. Thus, it remains unclear if TFMT treatment may induce inflammatory responses and affect cellular protein synthesis during longer durations of human flu treatment. Finally, it will also be important to explore the effects of TFMT under more realistic conditions, as flu patients typically begin treatment after symptom onset.
